# Integrating Frequency Guidance into Multi-Source Domain Generalization for Acoustic-Based Fault Diagnosis in Industrial Systems

**DOI:** 10.3390/s26092647

**Published:** 2026-04-24

**Authors:** Yu Wang, Hongyang Zhang, Yinhao Liu, Chenyu Ma, Xiaolu Li, Xiaotong Tu, Xinghao Ding

**Affiliations:** 1 School of Informatics, Xiamen University, Xiamen 361005, China; wangy070633@163.com (Y.W.); liuyinhao28@stu.xmu.edu.cn (Y.L.); machenyu@stu.xmu.edu.cn (C.M.); xttu@xmu.edu.cn (X.T.); dxh@xmu.edu.cn (X.D.); 2 School of Science and Engineering, The Chinese University of Hong Kong, Shenzhen 518172, China; hongyzhang@stu.xmu.edu.cn; 3 School of Science, Jimei University, Xiamen 361005, China

**Keywords:** acoustic fault diagnosis, domain generalization, frequency-domain augmentation

## Abstract

With the increasing demand for intelligent fault monitoring, acoustic-based diagnosis has emerged as a promising solution for industrial applications such as pipeline leakage and electrical equipment fault detection. However, complex working conditions and domain shifts significantly degrade model performance, especially when unseen target domain data is unavailable. To address this, we propose an amplitude-phase collaborative augmentation network named AP-CANet tailored for acoustic fault diagnosis. Specifically, the network adaptively aligns amplitude and phase features across multiple source domains and performs label-consistent sample augmentation to enrich data diversity while preserving semantic consistency. A frequency–spatial interaction module further integrates global spectral information with local temporal details to improve feature discriminability. Moreover, we introduce a manifold triplet loss that scales shortest path distances in the feature manifold, encouraging the model to better capture subtle distinctions among hard samples and improving intra-class compactness and inter-class separability. We evaluate the proposed method on two publicly available datasets: the Pipeline Leak Acoustic Dataset (GPLA-12) and the Electrical Sound Dataset (MIMII-DG). Experimental results demonstrate superior performance under domain-shift scenarios, highlighting the method’s potential for scalable and low-cost acoustic fault diagnosis in real-world industrial environments.

## 1. Introduction

Acoustic-based fault diagnosis has increasingly leveraged frequency-domain representations to capture the spectral characteristics of industrial signals [1,2,3,4,5]. However, unlike vibration signals, acoustic data is highly susceptible to non-stationary environmental noise, leading to significant domain shifts that degrade model generalization. While frequency-domain augmentation has gained momentum owing to its ability to manipulate spectral components [6,7,8], a critical gap remains: most current techniques rely on simple linear interpolation [9] or keep phase information fixed while only exchanging amplitude. Such approaches often fail to capture the intrinsic decoupling of amplitude (energy) and phase (structure) [10], resulting in ’semantic drift’, where fault-specific features are inadvertently altered. To address these challenges, this paper proposes a novel Amplitude-Phase Collaborative Augmentation Network (AP-CANet). Motivated by the need for label-consistent diversification, AP-CANet collaboratively leverages both amplitude and phase features across multiple source domains to learn robust representations. Our method offers distinct benefits over existing DG frameworks by introducing a Frequency–Spatial Interaction Module (FSIM) to bridge global spectral cues with local patterns, alongside a manifold triplet loss that reinforces category boundaries in a non-Euclidean manifold space. This problem-driven design ensures that the augmented features remain category-invariant while suppressing domain-specific interference.

Despite the success of Fourier-based augmentation, its application to industrial acoustics is limited by two gaps. First, it ignores physical decoupling where environmental noise affects amplitude while fault transients reside in phase, leading to “semantic drift” during random mixing; second, it lacks an adaptive mechanism to align these frequency components across domains. To bridge these gaps, our AP-CANet employs problem-driven Amplitude-Phase Collaborative Augmentation (APCA). By performing label-consistent augmentation and frequency–spatial interaction, APCA actively exploits the complementary nature of amplitude and phase to suppress domain interference while preserving category-invariant features.

The rest of this paper is organized as follows: Section 3 details the proposed AP-CANet architecture, Section 4 presents the experimental results and ablation analysis, and Section 5 concludes the study. The main contributions of this paper are summarized as follows:

- We propose a novel framework named AP-CANet that leverages the complementary properties of amplitude and phase spectra to perform multi-source frequency-domain augmentation for robust acoustic fault diagnosis.
- We design the FSIM module, which fuses global spectral and local spatial features in both amplitude and phase subspaces, enhancing feature discriminability and cross-domain transferability.
- We introduce a manifold triplet loss with a non-linear distance metric to improve the model’s ability to mine hard samples and learn discriminative representations under domain-shift conditions.
- We conduct comprehensive experiments on two public acoustic datasets (GPLA-12 and MIMII-DG), and the results demonstrate that AP-CANet outperforms state-of-the-art domain generalization methods in unseen target domains.

## 2. Related Works

### 2.1. Transfer Learning in Acoustic Fault Diagnosis

In recent years, transfer learning, especially domain adaptation (DA), has been widely applied in acoustic fault diagnosis to address the performance degradation caused by domain shifts under variable working conditions. Traditional models trained in specific environments often fail when applied to unseen domains due to differences in load, speed, and background noise. To overcome this, Xie et al. [11] proposed a hierarchical adversarial multi-target domain adaptation method using raw acoustic signals, which aligns multi-level features across domains and achieves robust gear fault diagnosis under complex conditions. Brusa et al. [12] argued that networks pre-trained on music recognition and sound detection can be leveraged for fault diagnosis and proposed YAMNet to achieve task transfer. Hasan et al. [13] proposed the use of acoustic spectral imaging (ASI) technology based on acoustic emission (AE) signals to visualize spectral features, ensuring their applicability in transfer learning. Yang et al. [14] further proposed a cross-domain acoustic diagnosis framework based on vibration–acoustic migration learning, which enhances generalization by aligning acoustic features with vibration-guided latent space.

While these works have achieved promising results, most have focused on spatial or hybrid spatial–temporal features, often overlooking the rich and generalizable structures encoded in the frequency domain. This underexplored area offers substantial potential, as frequency components often carry critical information about machinery faults, especially under variable noise levels. To this end, recent research has begun incorporating Fourier-based domain augmentation and frequency-disentanglement strategies to better utilize spectral characteristics for fault generalization. Nevertheless, current domain generalization studies with respect to acoustic fault diagnosis remain limited in scope, especially in modeling domain-invariant representations under noisy and highly dynamic conditions. This highlights the urgent need for more robust and frequency-aware DG frameworks tailored for real-world industrial environments.

### 2.2. Frequency-Domain Augmentation for Domain Generalization in Acoustic Fault Diagnosis

Early studies [10,15] demonstrated a key property of the Fourier transform: the phase spectrum encodes high-level semantic information, while the amplitude spectrum captures low-level statistical properties of the signal. In the context of acoustic fault diagnosis, this property is particularly valuable, as sound signals often exhibit rich frequency structures that reflect fault characteristics under different working conditions. Inspired by developments in computer vision, Kulevome et al. [16] proposed a data augmentation method based on STFT time-frequency data, which enhances sample diversity through perturbation and reconstruction of the time–frequency representation, thereby improving the robustness and generalization ability of fault diagnosis models under low-sample-size conditions. Xu et al. [9] further introduced a Fourier-based augmentation strategy via amplitude interpolation, enhancing phase-aware generalization through spectral mix-up. Lin et al. [17] explored deep models’ frequency bias and designed Deep Frequency Filtering (DFF) to extract components of varying domain-transfer difficulties in the latent space.

Recent research has begun to integrate frequency-domain information into domain-generalizable acoustic fault diagnosis. For instance, Liu et al. [18] introduced a Fourier-guided latent diffusion model that uses amplitude spectra to condition the generation of domain-invariant representations across machine types. Additionally, Wang et al. [19] developed a convolutional LSTM model processing time–frequency acoustic features of wind turbine bearings and achieved over 99.5% accuracy, demonstrating enhanced cross-domain generalization via spectral information. However, these works primarily limit the use of the Fourier transform to input-level preprocessing, without fully exploiting the complementary roles of amplitude and phase. To address this gap, we propose AP-CANet, which performs frequency-domain augmentation across both amplitude and phase spectra. By integrating manifold-aware metric learning, our method enhances the discriminability and robustness of learned representations, significantly improving fault diagnosis performance under unseen acoustic conditions.

## 3. Method

### 3.1. Motivation and Background

In acoustic fault diagnosis, the data distribution is highly sensitive to variations in industrial factors such as the working load, vibration frequency, and background noise. These variations lead to domain shifts that significantly degrade diagnostic performance. While frequency-domain analysis has been shown to alleviate such shifts, prior research has seldom explored discriminative feature extraction from frequency-transformed acoustic signals. To bridge this gap, we propose AP-CANet, which reconstructs both amplitude and phase representations in the Fourier domain. This enables the model to learn more robust, generalizable features that capture the underlying structure of acoustic signals across diverse working conditions.

First, we revisit the definition and properties of the Fourier transform. The Fourier transformation (F(x)) a signal (*x*) with a shape of C×H×W is formulated as follows:

(1) $$\begin{array}{cc} F(x)(c,u,v) & =X(c,u,v)\\ \; & =\sum_{h=0}^{H-1}\sum_{w=0}^{W-1}x\left(c,h,w\right)e^{-j2\pi (\frac{h}{H}u+\frac{w}{W}v)}, \end{array}$$

where *C*, *H*, and *W* are the channel, height, and width values of the signal, respectively.

Let X(f) denote the complex-valued Fourier transform of the acoustic signal (x(t)). We define the amplitude spectrum (A(f)) and phase spectrum (P(f)) as follows:

(2) $$\begin{array}{cc} \; & A\left(X\left(c,u,v\right)\right)=\sqrt{R^{2}\left(X\left(c,u,v\right)\right)+I^{2}\left(X\left(c,u,v\right)\right)},\\ \; & P\left(X\left(c,u,v\right)\right)=\arctan\left[\frac{I(X(c,u,v))}{R(X(c,u,v))}\right], \end{array}$$

where R(x) and I(x) denote the real and imaginary parts of X(c,u,v). After decoupling the amplitude and phase, we perform label-consistent augmentation. The augmented frequency-domain feature ($\hat{X}$) is formulated as follows:

(3) $$\hat{X}=\left[\left(1-\lambda \right)A\left(X_{i}\right)+\lambda A\left(X_{j}\right)\right]\cdot e^{j[\left(1-\eta \right)P\left(X_{i}\right)+\eta P\left(X_{j}\right)]},$$

where $X_{i}$ and $X_{j}$ denote the complex frequency-domain representations of two different samples from the source domains. λ and η are mixing coefficients sampled from a Dirichlet or Beta distribution that control the fusion ratio of the amplitude (style-related) and phase (structure-related) components. Finally, the augmented feature in the spatial domain is obtained by applying the Inverse Fast Fourier Transform (IFFT): $\hat{x}=\mathrm{IFFT}\left(\hat{X}\right)$.

According to Fourier theory, the amplitude component (A) reflects the stylistic characteristics of the signal in the frequency domain, while the phase component (P) encodes structure-related information that is closely tied to the signal category. Empirical analysis reveals that amplitude varies significantly across domains, even within the same class, whereas the phase remains relatively consistent, indicating its robustness to domain shifts. Motivated by this observation, we propose the enhancement of intra-class diversity and cross-domain consistency by progressively extracting and reconstructing both amplitude and phase components. This strategy enables the model to learn more generalized representations, thereby reducing domain discrepancies and improving robustness under unseen conditions.

### 3.2. AP-CANet

**Figure 1:**
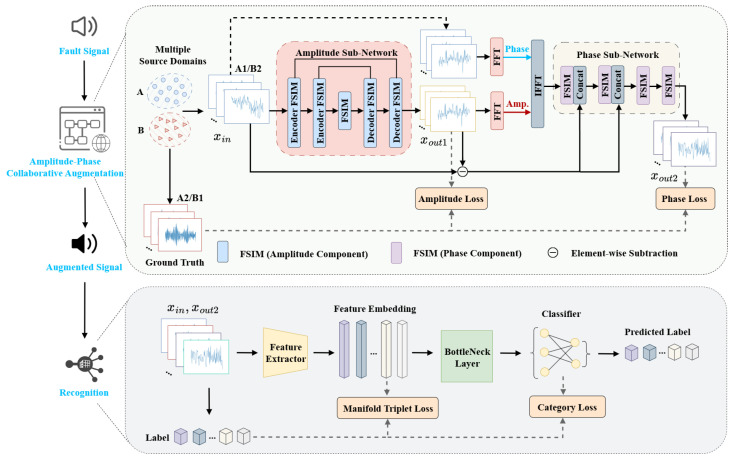
The overall architecture of AP-CANet.

Based on the above analysis, we introduce a simple yet effective AP-CANet framework, as shown in Figure 1. The overall network consists of an amplitude sub-network and a phase sub-network, both of which are designed to generate augmented amplitude and phase representations. Each sub-network incorporates a frequency–spatial interaction module (FSIM) as a fundamental building block, which integrates global and local spatial information to enhance feature extraction and learning.

In this process, a subset ($A_{1}$) from domain *A* is selected as an input feature. AP-CANet adaptively aligns the amplitude and phase representations to the corresponding features ($B_{1}$) in domain *B*. Meanwhile, another sample subset ($B_{2}$) from domain *B* is used as the target, and AP-CANet aligns amplitude and phase components to approach subset $A_{2}$ in domain *A*. This bidirectional cross-domain matching enhances feature consistency and improves model generalization under diverse domain conditions. We take the alignment from $A_{1}$ to $B_{1}$ as an example to describe the processing flow of the two sub-networks in AP-CANet.

In the amplitude sub-network, a sample from $A_{1}$ is selected as input ($x_{in}$) and is passed through five FSIM-based encoder–decoder blocks to generate the output feature ($x_{out1}$). Meanwhile, the corresponding sample ($x_{gt}$) from $B_{1}$ under the same category is used as the target. In amplitude space, the three signals ($x_{in}$, $x_{gt}$, and $x_{out1}$) form a constraint. To guide the learning of amplitude components, we extract the amplitude spectrum ($A(x_{gt})$) as a soft label and constrain the output ($X_{out1}$). The amplitude loss ($L_{amp}$) is defined as follows:

(4) $$X_{out1}=F\left(G_{A}\left(x_{in}\right)\right),$$

(5) $$L_{amp}={∥A\left(X_{out1}\right)-A\left(x_{gt}\right)∥}_{1},$$

where ${∥\cdot ∥}_{1}$ denotes the mean absolute error, $G_{A}$ is the amplitude sub-network, F is the Fourier transform operator, and A(·) represents amplitude extraction from the spectrum.

In the phase sub-network, we use four FSIM blocks specialized for phase representation learning. The first two blocks receive residual information from the amplitude sub-network to preserve cross-modal consistency. To ensure consistency of the input, we do not feed $x_{out1}$ directly into the phase sub-network. Instead, we use the reconstructed amplitude ($F^{-1}\left(A\left(x_{out1}\right)\right)$) and the original phase ($P(x_{in})$) as inputs:

(6) $$x_{out2}=F\left(G_{P}\left(F^{-1}\left(A\left(x_{out1}\right),P\left(x_{in}\right)\right)\right)\right).$$

To recover time-domain signals, we adopt the Inverse Fast Fourier Transform (IFFT) for signal reconstruction, treating the processed signals as complexly valued inputs. Considering the sensitivity of phase components to structural and environmental changes in real-world scenarios, we further model the residual phase differences between $x_{in}$ and $x_{out1}$ to guide alignment. In implementation, the residual features are concatenated with $P(x_{in})$, followed by a 1×1 convolution for integration. The phase loss is defined as follows:

(7) $$L_{pha}={∥P\left(x_{out2}\right)-P\left(x_{gt}\right)∥}_{1},$$

where $G_{P}$ is the phase sub-network and P(·) represents the phase extraction function. Finally, the total augmentation loss of AP-CANet is computed as follows:

(8) $$L_{aug}=\lambda_{1}L_{amp}+\lambda_{2}L_{pha},$$

where $\lambda_{1}$ and $\lambda_{2}$ are weighting factors, which, in this paper, we set to 0.2 and 0.4, respectively.

The selection of AP-CANet is driven by the need to decouple amplitude (style) and phase (structure), a capability standard spatial-domain CNNs lack but that is vital for acoustic domain generalization. By bridging global spectral cues and local patterns via FSIM, the model achieves superior robustness. While the dual-branch structure and FFT operations increase computational complexity, this overhead is justified by the significant gains in diagnostic reliability under varying industrial conditions, offering a more resilient solution than traditional single-stream architectures.

### 3.3. Frequency–Spatial Interaction Module

According to the theoretical analysis in [20], frequency-domain operations allow models to capture global representations, while convolutional layers are mainly focused on extracting local spatial features. Inspired by this observation, we introduce the Frequency–Spatial Interaction Module (FSIM) as a fusion block in both the amplitude and phase sub-networks to enhance their joint representation learning capabilities.

**Figure 2:**
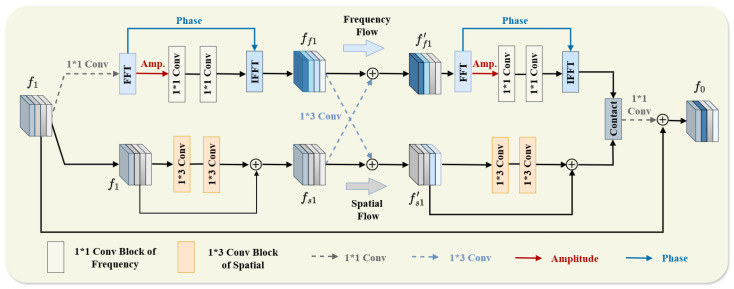
The overall architecture of FSIM.

As illustrated in Figure 2, FSIM consists of two branches: a frequency branch and a spatial branch. First, the input source feature ($f_{1}$) is fed into FSIM. In the spatial branch, a series of 1×3 convolutional residual blocks are applied to extract spatial information, producing output $f_{s1}$.

Simultaneously, the frequency branch processes $f_{1}$ using a 1×1 convolution to obtain $f_{f0}$, which is then transformed into the frequency domain via the Fourier transform, yielding spectrum representation $F_{f0}$. Amplitude and phase components are constructed in the frequency domain. To further extract amplitude information, we introduce a convolutional encoder module. The final output of the frequency branch is reconstructed via inverse Fourier transform as follows:

(9) $$f_{f1}=F^{-1}\left({\mathrm{Conv}}_{1\times 1}\left(A\left(F_{f0}\right)\right),P\left(F_{f0}\right)\right),$$

where ${\mathrm{Conv}}_{1\times 1}$ denotes the 1×1 convolution operation.

Next, the two branch outputs ($f_{f1}$ and $f_{s1}$) are passed through 1×3 convolution layers to encourage feature interaction between the two branches. The updated features are calculated as follows:

(10) $$\begin{array}{cc} f_{f1}^{\prime } & =f_{f1}+{\mathrm{Conv}}_{1\times 3}\left(f_{s1}\right),\\ f_{s1}^{\prime } & =f_{s1}+{\mathrm{Conv}}_{1\times 3}\left(f_{f1}\right), \end{array}$$

where ${\mathrm{Conv}}_{1\times 3}$ denotes the 1×3 convolution operation.

As shown in Figure 2, the outputs ($f_{f1}^{\prime }$ and $f_{s1}^{\prime }$) capture complementary features through interaction, enhancing model discriminability. This process is repeated in subsequent FSIM layers. The final output of FSIM is denoted as $f_{f0}$. Similarly, in the phase branch, the amplitude function (A(·)) is replaced by the phase function (P(·)), while the remaining operations remain unchanged.

### 3.4. Manifold Triplet Loss

In traditional machine learning, it is typically assumed that the distance between samples reflects their similarity in Euclidean space, where data is linearly and uniformly distributed. However, in real-world applications such as rotating machinery fault diagnosis, the acquired signals are often affected by installation, environmental, and operating conditions, resulting in non-linear and complex data distributions that are hard to model using Euclidean assumptions.

**Figure 3:**
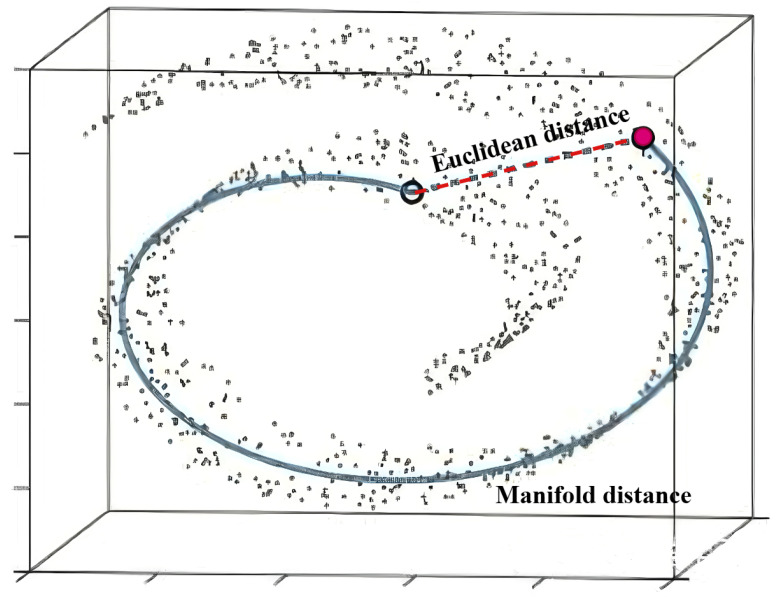
Manifold space distance metric.

Manifold space refers to a geometric space that appears Euclidean locally but may exhibit curved structure globally. As shown in Figure 3, Euclidean distances fail to reflect true sample similarity paths, whereas manifold distances measured along the curved data structure preserve more intrinsic relationships. Motivated by this, we propose a manifold triplet loss that captures intra-class and inter-class relationships via semantic distances on the manifold.

To model this, we replace Euclidean distance with shortest path distance on a manifold graph. Specifically, a graph is constructed where each node represents a sample, and edges connect to its *K* nearest neighbors. The edge weights are defined by pairwise local Euclidean distances. A sparse adjacency matrix (*D*) is built such that

(11) D(i,j)=min(D(i,j),D(i,k)+D(k,j)),

where D(i,j) is the shortest path between nodes *i* and *j* and *k* denotes any intermediate node. The Floyd–Warshall algorithm is used to compute shortest paths among all sample pairs.

To emphasize the importance of hard boundary samples, we define a nonlinear distance rescaling function:

(12) $$d_{m}\left(D\left(i,j\right)\right)=\left\{\begin{array}{cc} k\cdot D(i,j), & D(i,j)>r\\ D(i,j)/k, & D(i,j)\leq r \end{array}\right.$$

where *r* is the mean intra-class distance in the current mini-batch and *k* is a scaling coefficient controlling the strength of expansion and contraction (set to 3). Finally, the manifold triplet loss is defined as follows:

(13) $$L_{mt}=\max\left(d_{m}^{p}\left(x\right)-d_{m}^{n}\left(x\right)+\gamma ,\;0\right),$$

where $d_{m}^{p}\left(x\right)$ and $d_{m}^{n}\left(x\right)$ denote the manifold distances of the hardest positive and negative pairs within a mini-batch and γ is the margin. This approach enables the model to better capture intra-class cohesion and inter-class separation in non-Euclidean data structures.

### 3.5. Loss Function

During the classification stage, both the original input ($x_{in}$) and the augmented sample ($x_{out2}$) are used to construct the training dataset to enhance the model’s ability to identify different fault types. The softmax function is used to calculate the predicted probability ($p(v_{i})$), which is defined as follows:

(14) $$p\left(v_{i}\right)=\frac{e^{v_{i}}}{\sum_{j=1}^{N_{cls}}e^{v_{j}}},\;i=1,2,...,N_{cls}$$

where $v_{i}$ represents the logit score of the *i*-th fault instance and $N_{cls}$ denotes the number of fault classes. The prediction with the highest probability is selected as the final classification result. The model is optimized by minimizing the cross-entropy loss between the predicted results and the ground truth:

(15) $$L_{clf}=-\sum_{i=1}^{N_{cls}}\log\left(p\left(v_{i}\right)\right).$$

By combining all loss terms, the total training objective of AP-CANet is expressed as

(16) $$L_{total}=L_{aug}+L_{clf}+\alpha L_{mt},$$

where α is a weighting coefficient used to balance the contribution of the manifold triplet loss.

## 4. Experiment

To verify the effectiveness and generalization capability of the proposed AP-CANet in acoustic fault diagnosis, comprehensive experiments are conducted on two publicly available acoustic fault diagnosis datasets: the Pipeline Leak Acoustic Dataset (GPLA-12) and the Electrical Sound Dataset (MIMII-DG). These datasets cover a variety of operating conditions and simulate domain shifts commonly encountered in industrial environments.

All models are trained using the same backbone network for fairness, and performance is measured using classification accuracy, confusion matrix analysis, and feature visualization (e.g., t-SNE). Furthermore, ablation studies are conducted to investigate the contributions of amplitude-phase augmentation and manifold-aware metric learning.

### 4.1. Dataset Description

The inclusion of the MIMII-DG dataset alongside GPLA-12 is intended to evaluate the model’s cross-scenario generalization. While GPLA-12 focuses on localized pipeline leakages, MIMII-DG provides acoustic data from rotating industrial fans under varying SNR conditions. By achieving consistent performance across these two fundamentally different acoustic environments, we demonstrate that APCANet is not overfit to a specific hardware setup but is robust to diverse industrial soundscapes.

**Figure 4:**
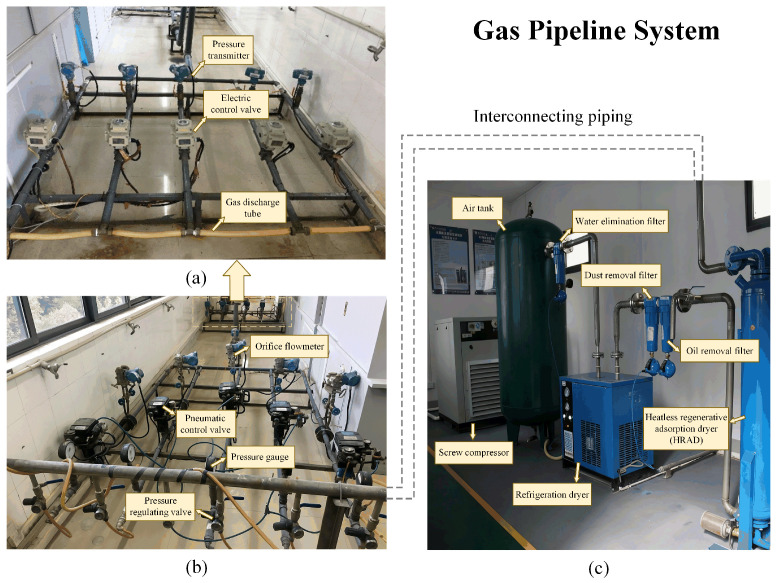
Illustration of the gas pipeline system. (**a**) The back end of gas pipeline system; (**b**) the front end of gas pipeline system; (**c**) air compressor units.

GPLA-12 [21]: GPLA-12 is an open-source acoustic dataset specifically designed for gas pipeline leak detection. The real gas pipeline system with detailed components shown in Figure 4 is used to collect the GPLA-12 dataset. The dataset consists of 684 samples categorized into 12 classes, each representing different leakage intensities, pressure levels, and background noise conditions. To evaluate the generalization performance of the proposed method under domain-shift scenarios, we divide the dataset into domains based on sound pressure levels (SPLs), simulating realistic acoustic variations caused by changes in environmental energy. Specifically, the dataset is partitioned into three domains corresponding to pressure levels of 0.2 MPa, 0.4 MPa, and 0.5 MPa (expressed as $GP_{1}$, $GP_{2}$, and $GP_{3}$), with each domain containing four distinct classes. Each sample is a one-dimensional acoustic signal of size 1×1×1460.

**Figure 5:**
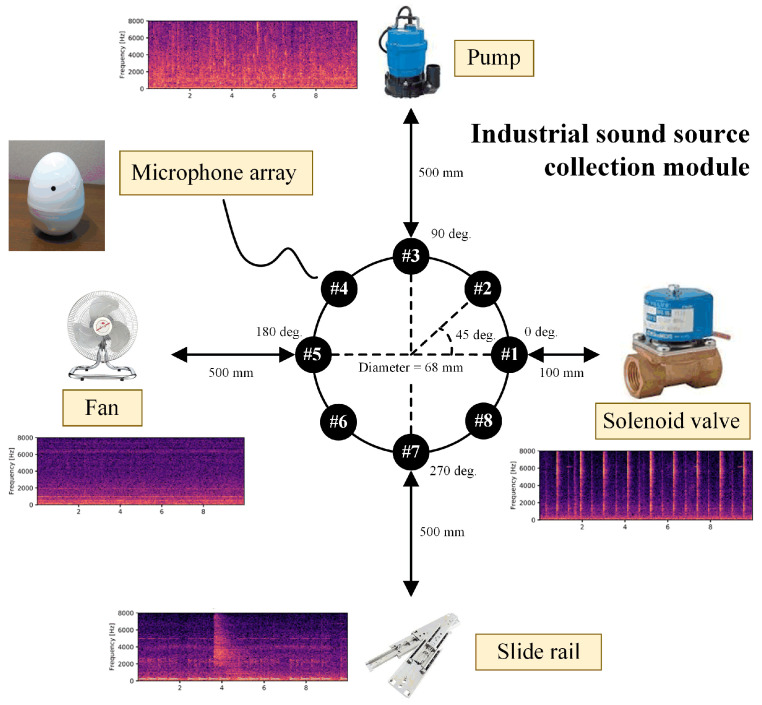
Schematic diagram of an industrial equipment sound-source collection setup. This includes four typical industrial machines (pump, fan, solenoid valve, and slide rail) with their power spectrogram samples under normal conditions at 6-dB SNR.

MIMII-DG [22]: The MIMII-DG dataset is designed for acoustic monitoring of industrial equipment including pumps, fans, solenoid valves, and slide rails, containing machine operation sounds under varying background noise conditions. Figure 5 depicts the recording setup for each machine’s orientation and distance relative to the microphone array. In this study, we adopt a domain-partitioning strategy based on noise levels, using varying sound pressure levels (SPLs) to simulate different noise environments: −6 dB, 0 dB, and 6 dB (expressed as $MI_{1}$, $MI_{2}$, and $MI_{3}$). Each domain includes two classes: normal and anomalous, with anomalies covering faults such as leakage, contamination, and component damage. Experiments are conducted using the fan device data. The model is trained on two source domains and evaluated on an unseen target domain, without accessing its data during training. Audio was recorded using an 8-channel microphone array at 16 kHz. Each sample has a shape of 8×1×3600.

As shown in Table 1, the GPLA-12 dataset consists of 12 classes recorded in three spatial domains representing different sensor-to-leak distances. The MIMII-DG dataset (fan subset) includes industrial fan sounds mixed with ambient factory noise at various signal-to-noise ratios (SNRs). To ensure reliability, we adopt a “leave-one-domain-out” strategy: the model is trained on two domains (with an 80/20 split for internal validation) and evaluated on the entirely unseen third domain to simulate real-world deployment.

The labeling procedure follows the physical configurations of the data collection process. For GPLA-12, each acoustic sample is assigned a label (y∈{0 , 1, ⋯, 11}) corresponding to 12 distinct leakage scenarios (varying by hole size and location). For MIMII-DG, we adopt a binary labeling scheme where ‘0’ represents normal operational sound and ‘1’ indicates anomalous fan behavior. All labels are cross-verified with the ground-truth mechanical states recorded during the experiments to ensure high data fidelity before the training phase.

### 4.2. Experimental Setting

Our proposed method is implemented using the PyTorch 2.1.0 framework [23] on Python 3.9 and a single NVIDIA 2080 Ti GPU. During the training process, the framework is trained for 50 epochs with a batch size of 128. The SGD optimizer [24] is employed to update the overall framework with a momentum of 0.9. Due to the different sensitivity of the Amplitude-Phase Collaborative Augmentation Network, the initial learning rates are set to 0.001 and 0.01. To train the manifold triplet loss, each batch is constructed by a PK sampler [25,26], which comprises four types, each of which is composed of 32 instances in a batch. The constant threshold (r) is calculated by the mean distance in a batch, and the margin is 0.3 in the manifold triplet loss. The value of α is set to 0.01 in the training procedure. To ensure the reliability and effectiveness of the experimental results, we conducted experiments for each method five times for each task.

To quantitatively evaluate the performance of AP-CANet, we employ accuracy and standard deviation as the primary metrics. Accuracy is defined as the ratio of correctly predicted samples to the total number of samples:

(17) $$\mathrm{Acc}=\frac{TP+TN}{TP+TN+FP+FN}\times 100\%$$

where TP, TN, FP, and FN represent true positives, true negatives, false positives, and false negatives, respectively. Since domain generalization involves multiple trials across different target domains, we also report the standard deviation (Std) of the accuracy to demonstrate the model’s stability and reliability. A high average accuracy combined with a low standard deviation indicates that the proposed model is not only efficient but also consistently robust against diverse acoustic domain shifts.

### 4.3. Comparative Experiment

In this experiment, we compared our proposed method with two categories of benchmark models: a classical DA method, i.e., MMD [27], and several widely adopted DG methods, including SNR [28], Mixup [29], and MixStyle [30]. The average classification accuracies and standard deviations of all methods are reported in Table 2.

Table 2 provides a comprehensive comparison of the domain generalization performance of various methods on two challenging acoustic datasets: GPLA-12 and MIMII-DG (fan device). The reported metrics are the average classification accuracy and standard deviation (mean ± std), which, together, reflect both predictive performance and stability across three cross-domain transfer tasks.

AP-CANet consistently outperforms all the compared methods on both datasets. On the GPLA-12 dataset, which involves complex inter-task generalization (e.g., $GP_{1,2}\to GP_{3}$), AP-CANet achieves the best results in all three transfer tasks, with an average accuracy of 81.09% and a standard deviation of only 1.86, indicating both superior recognition capability and stable model behavior under domain shift. On the MIMII-DG dataset, where the domain gap arises mainly from different background noise levels, AP-CANet, again, demonstrates excellent robustness. It achieves accuracies of 85.76%, 84.68%, and 82.41% across the three tasks ($M_{1,2}\to M_{3}$, $M_{1,3}\to M_{2}$, and $M_{2,3}\to M_{1}$), resulting in an average of 84.28% ± 1.74.

In contrast, the ResNet18 backbone exhibits the weakest generalization performance, with average accuracies of 68.07% ± 4.35 on GPLA-12 and 70.69% ± 2.61 on MIMII-DG, confirming its limited robustness under domain shift. The MMD method, which reduces domain discrepancies via distribution alignment, performs well on GPLA-12 (79.12% ± 1.89) but achieves only 80.90% ± 2.68 on MIMII-DG, indicating that its effectiveness may be limited in real-world noisy scenarios. The SNR-based method benefits from explicit modeling of the signal-to-noise ratio, which aligns with the domain definitions in MIMII-DG, yielding 79.77% ± 2.57. However, its performance drops to 76.73% ± 2.57 on GPLA-12, reflecting limited adaptability to more abstract domain shifts.

Feature-level augmentation methods such as Mixup and MixStyle show moderate performance. Mixup achieves 72.04% and 78.00% average accuracies on GPLA-12 and MIMII-DG respectively, while MixStyle reaches accuracies of 70.98% and 78.80%. Despite occasional improvements, their relatively large standard deviations (e.g., MixStyle’s 3.49 on GPLA-12) indicate inconsistency and potential sensitivity to varying domain compositions.

**Figure 6:**
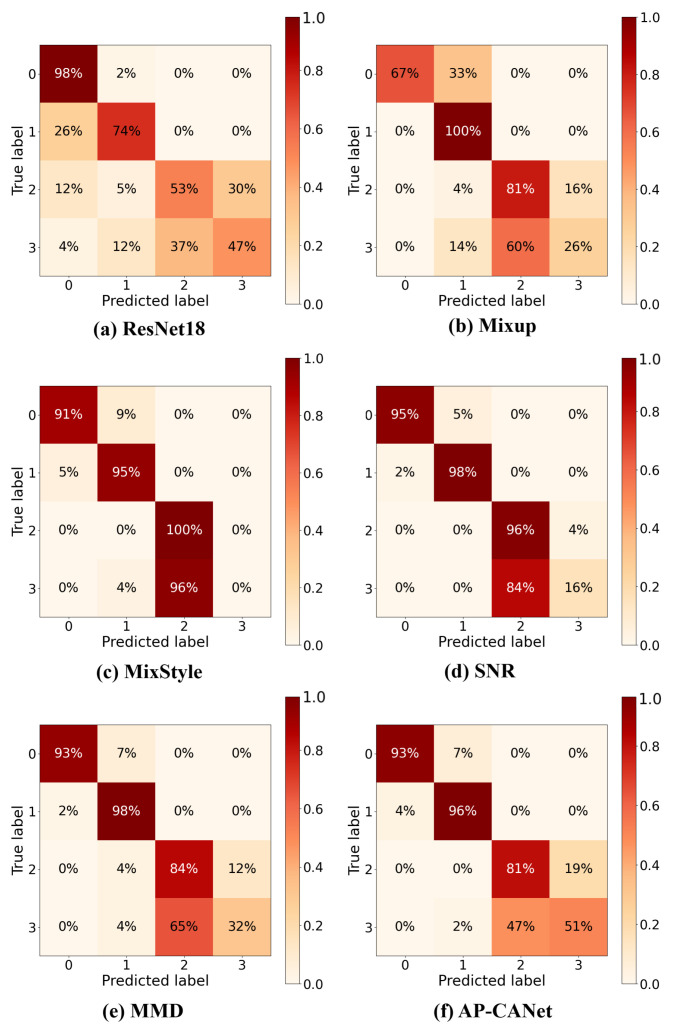
Confusion matrix results of different methods on the GPLA-12 dataset.

As shown in the confusion matrices in Figure 6, AP-CANet achieves the best overall performance on the GPLA-12 dataset, with over 90% accuracy in three out of four classes and the lowest overall misclassification rate. This demonstrates its superior generalization ability and stability across tasks. In contrast, ResNet18 and Mixup suffer from severe misclassification between classes 2 and 3. MixStyle and SNR show some improvements but still exhibit class boundary ambiguity. MMD performs poorly in distinguishing class 3. Overall, AP-CANet exhibits stronger discriminative capability and robustness under complex acoustic conditions.

Overall, AP-CANet leverages amplitude-phase collaborative augmentation to effectively capture both amplitude-invariant and phase-sensitive representations in the frequency domain. This design enables robust and accurate fault detection under diverse domain shifts, demonstrating strong generalization capability across both synthetic and real-world acoustic scenarios.

### 4.4. Discussion and Implications

The experimental results across the GPLA-12 and MIMII-DG datasets yield several major findings. First, the consistent superiority of AP-CANet confirms that frequency-domain decoupling is more effective for acoustic signals than standard spatial-domain augmentations. As noted in [10], phase information encodes the essential structural patterns of acoustic wavefronts. By preserving phase consistency while diversifying amplitude, our model successfully mitigates domain shift without losing fault-specific semantics.

Second, the performance gain in low-SNR scenarios (MIMII-DG) suggests that the FSIM module effectively filters environmental noise by bridging global spectral trends with local temporal transients. This aligns with the observations in [5] regarding the importance of multi-scale feature interaction in complex soundscapes. The implication for industrial practice is significant: AP-CANet reduces the need for extensive manual data re-labeling when deploying diagnostic systems to new factories with different background noise levels.

Finally, the success of the manifold triplet loss indicates that mapping acoustic features to a non-Euclidean manifold space better captures the underlying geometry of fault distributions than traditional Euclidean metrics. This finding reinforces the theory that high-dimensional acoustic data often resides on low-dimensional manifolds, as discussed in recent DG literature. In summary, these results demonstrate that integrating acoustic physical priors (amplitude phase) with advanced metric learning provides a more reliable and interpretable pathway for robust machine health monitoring.

### 4.5. Ablation Experiments on Core Modules

To verify the effectiveness of the proposed components in AP-CANet, we conduct a detailed ablation study on the GPLA-12 dataset. The results are summarized in Table 3, where we systematically evaluate the contribution of Amplitude-Phase Collaborative Augmentation (APCA), the two sub-branches (amplitude (amp) and phase (pha)) of the Frequency–Spatial Interaction Module (FSIM), and the Manifold Triplet Loss (MTL).

(1) Effectiveness of APCA: The baseline model (without any proposed modules) achieves an average accuracy of 75.82%. With the incorporation of APCA, the performance increases to 78.16%. This improvement confirms that frequency-guided augmentation effectively addresses the domain-shift problem by enriching the diversity of the training samples while preserving their semantic consistency.
(2) Contribution of FSIM Branches: We further investigate the impact of the dual-branch interaction within the FSIM. Adding only an amplitude branch (amp) or phase branch (pha) to the augmented baseline improves the accuracy to 78.68% and 79.06%, respectively. The best intermediate result (80.35%) is achieved when both branches are integrated, demonstrating that the collaborative interaction between global spectral information and local spatial details is essential for the extraction of discriminative features that are robust to environmental noise.
(3) Impact of MTL: The inclusion of MTL as the final constraint brings the overall performance to its peak of 81.09%. This incremental gain validates that our manifold-based metric learning effectively refines the feature space by narrowing intra-class distances on the data manifold, providing superior generalization to unseen target domains.

In conclusion, the ablation results demonstrate a clear performance ladder, confirming that each design choice—from data-level augmentation to architectural feature fusion and loss-level regularization—plays a distinct and vital role in the success of AP-CANet.

### 4.6. Ablation Study on the Hyperparameters of the Augmentation Network

**Figure 7:**
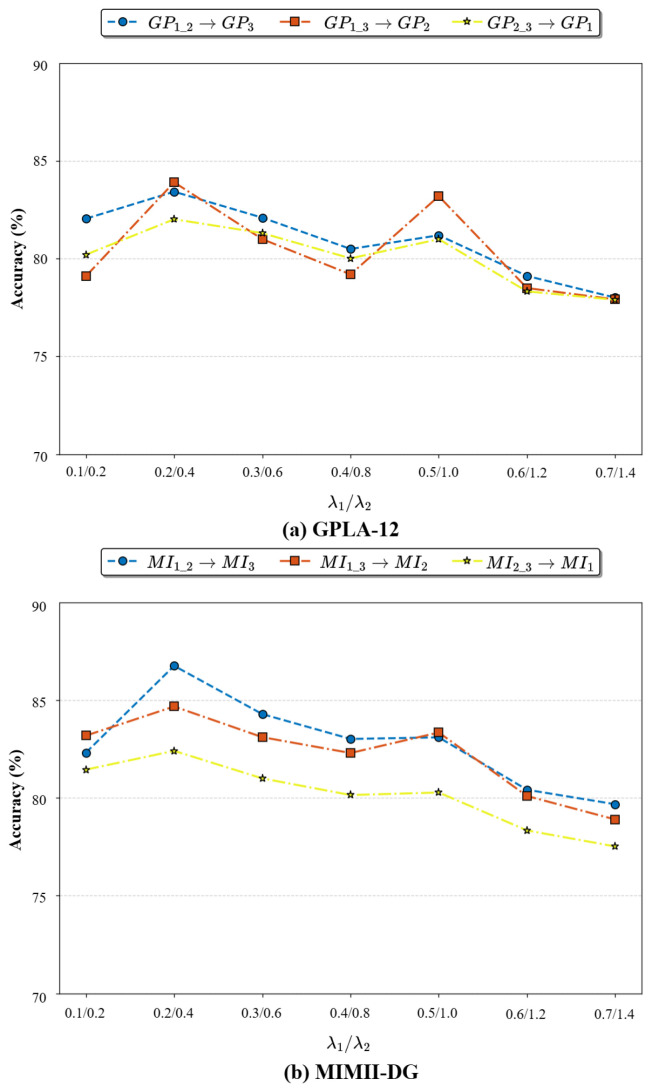
Line chart of the ablation study on regularization weight factors ($\lambda_{1}$ and $\lambda_{2}$).

To systematically evaluate the impact of the regularization weight factors ($\lambda_{1}$ and $\lambda_{2}$) on model performance, this study includes an ablation experiment using a set of proportional parameter combinations: $\lambda_{1}/\lambda_{2}\in$ (0.1/0.2, 0.2/0.4, 0.3/0.6, 0.4/0.8, 0.5/1.0, 0.6/1.2, 0.7/1.4). As shown in Figure 7, when $\lambda_{1}/\lambda_{2}=0.2/0.4$, the model achieves the highest diagnostic accuracies of 83.91% on the GPLA-12 dataset and 86.76% on the MIMII-DG dataset across different sub-tasks. Compared to the second-best combination ($\lambda_{1}/\lambda_{2}=0.5/1.0$), this setting improves accuracy by 1.70 and 3.66 percentage points, respectively.

In the low-parameter range ($\lambda_{1}<0.2$), insufficient regularization leads to model overfitting, causing the GPLA-12 test accuracy to drop to 79.11%. Conversely, in the high-parameter range ($\lambda_{1}>0.4$), excessive regularization results in feature degradation, with the MIMII-DG accuracy dropping sharply by 6.76%. Notably, maintaining a 1:2 ratio between $\lambda_{1}$ and $\lambda_{2}$ enables a dynamic balance between the amplitude and phase sub-networks, effectively promoting multi-source domain-feature learning and augmentation and optimizing cross-domain feature disentanglement.

### 4.7. Industrial Applications

The proposed AP-CANet architecture offers significant practical value for modern industrial systems. Specifically, it can be deployed in smart manufacturing plants for the continuous health monitoring of rotating components (e.g., industrial fans and pumps), where its robustness to environmental noise ensures high diagnostic accuracy without frequent recalibration. Furthermore, in gas pipeline infrastructure, the model can be integrated into automated leak detection systems to provide real-time alerts, potentially preventing costly energy losses and environmental hazards. By leveraging the model’s domain generalization capability, enterprises can significantly reduce the costs associated with data acquisition and expert labeling when scaling diagnostic solutions across different geographic sites or equipment generations.

## 5. Conclusions

This paper addresses the challenge of domain generalization in acoustic fault diagnosis under complex working conditions and domain shifts, especially when target-domain data is unavailable. To tackle this, AP-CANet is proposed in this paper. The network adaptively aligns amplitude and phase features across multiple source domains and introduces a frequency–spatial interaction module, along with a manifold triplet loss to enhance feature discrimination while maintaining semantic consistency. Experimental evaluations on the GPLA-12 and MIMII-DG datasets demonstrate that the proposed method achieves superior performance in unseen domains and under noisy conditions. Despite its performance, this study has limitations. First, the dual-branch interaction increases computational complexity, potentially hindering real-time deployment on edge devices. Second, the reliance on amplitude-phase decoupling may be less effective for signals with extreme non-stationary noise. Future work will focus on developing lightweight architectures and adaptive frequency selection. Additionally, extending AP-CANet to open-set scenarios—where unseen fault categories emerge in the target domain—remains a promising direction for the enhancement of industrial reliability.

In the long term, the principles of amplitude-phase collaborative interaction established in this study can provide a foundation for the development of more resilient acoustic diagnostic frameworks. Future research will explore the extension of AP-CANet to multimodal fusion scenarios, where acoustic data is combined with vibration or thermal imagery for more comprehensive system health assessment. Additionally, we aim to investigate self-supervised pre-training techniques to further reduce the dependency on labeled source-domain data, making the model more adaptable to extreme environments where fault samples are scarce. Ultimately, these advancements will contribute to the realization of truly autonomous and self-evolving industrial maintenance platforms.

## Figures and Tables

**Table 1:** Detailed statistics and experimental settings of the datasets.

Dataset	Machine/Fault Type	Domain Factor	Recording Conditions	Total Samples	Train/Val/Test Split
GPLA-12	12 (Leakage size & location)	Sensor distance	Lab (50 kHz sampling)	7200	80% Train/20% Val/100% Test
MIMII-DG	2 (Normal & abnormal fan)	Background noise	SNR: −6 dB, 0 dB, 6 dB	12,020	80% Train/20% Val/100% Test

**Table 2:** Comparison of domain generalization accuracy of different methods on the GPLA-12 and MIMII-DG datasets. The reported metrics are the average classification accuracy, along with standard deviation (mean ± std). The best results are marked in **bold**.

Method	GPLA-12 Dataset				MIMII-DG Dataset			
	${\mathrm{GP}}_{1\_2}$ → ${\mathrm{GP}}_{3}$	${\mathrm{GP}}_{1\_3}$ → ${\mathrm{GP}}_{2}$	${\mathrm{GP}}_{2\_3}$ → ${\mathrm{GP}}_{1}$	Avg	${\mathrm{MI}}_{1\_2}$ → ${\mathrm{MI}}_{3}$	${\mathrm{MI}}_{1\_3}$ → ${\mathrm{MI}}_{2}$	${\mathrm{MI}}_{2\_3}$ → ${\mathrm{MI}}_{1}$	Avg
ResNet18 [31]	67.30 ± 4.22	69.36 ± 4.66	67.54 ± 3.87	68.07 ± 4.35	74.84 ± 3.10	72.23 ± 3.48	70.69 ± 3.12	73.92 ± 3.23
MMD [27]	78.39 ± 1.50	80.34 ± 2.50	78.62 ± 1.67	79.12 ± 1.89	82.41 ± 2.32	80.92 ± 2.78	79.36 ± 2.94	80.90 ± 2.68
SNR [28]	71.35 ± 3.08	74.83 ± 2.54	73.22 ± 2.83	73.13 ± 2.82	81.14 ± 2.63	80.35 ± 2.21	77.81 ± 2.87	79.77 ± 2.57
MIXUP [29]	69.07 ± 2.99	70.15 ± 3.28	69.82 ± 1.87	69.68 ± 2.71	78.79 ± 2.51	79.66 ± 2.86	77.94 ± 2.75	78.80 ± 2.71
MIXSTYLE [30]	69.92 ± 4.42	72.19 ± 3.95	70.83 ± 2.09	70.98 ± 3.49	78.60 ± 2.32	80.47 ± 2.12	77.33 ± 2.19	78.80 ± 2.21
**AP-CANet (ours)**	**80.93 ± 1.47**	**82.02 ± 2.46**	**80.31 ± 1.64**	**81.09 ± 1.86**	**85.76 ± 1.84**	**84.68 ± 1.65**	**82.41 ± 1.72**	**84.28 ± 1.74**

**Table 3:** Results of ablation experiments on the core modules on the GPLA-12 dataset. The reported metrics are the average classification accuracy and standard deviation (mean ± std). The best results are marked in **bold**.

APCA	FSIM		MTL	GPLA-12 Dataset			
	amp	pha		${\mathrm{GP}}_{1\_2}$ → ${\mathrm{GP}}_{3}$	${\mathrm{GP}}_{1\_3}$ → ${\mathrm{GP}}_{2}$	${\mathrm{GP}}_{2\_3}$ → ${\mathrm{GP}}_{1}$	Avg
✗	✗	✗	✗	75.01 ± 1.98	78.14 ± 2.23	74.31 ± 1.84	75.82 ± 2.02
✓	✗	✗	✗	78.16 ± 1.66	79.98 ± 2.01	76.33 ± 1.53	78.16 ± 1.73
✓	✓	✗	✗	78.66 ± 1.65	79.39 ± 1.91	77.98 ± 2.21	78.68 ± 1.92
✓	✗	✓	✗	78.17 ± 1.87	80.09 ± 1.88	78.92 ± 1.99	79.06 ± 1.91
✓	✓	✓	✗	79.35 ± 1.52	81.93 ± 1.58	79.76 ± 1.89	80.35 ± 1.66
✓	✓	✓	✓	**80.93 ± 1.47**	**82.02 ± 2.46**	**80.31 ± 1.64**	**81.09 ± 1.86**

## Data Availability

The data used to support the findings of this study are available from the corresponding author upon request.
